# Exploring micro-CT as a novel approach for postmortem fingerprint identification

**DOI:** 10.1007/s00414-026-03760-x

**Published:** 2026-03-09

**Authors:** Greta M.M. Timmerman, Daniel Docter, Bart L.M. Kraus, Jermo Hanemaaijer-van der Veer, Roelof-Jan Oostra, Bernadette S. De Bakker

**Affiliations:** 1 Dept. of Obstetrics and Gynecology, Amsterdam UMC location University of Amsterdam, Amsterdam, The Netherlands; 2 Netherlands School of Public & Occupational Health (NSPOH), Forensic Medicine, Utrecht, The Netherlands; 3 Dept. of Pediatric Surgery, Amsterdam UMC, location AMC, Emma Children’s Hospital, Amsterdam, The Netherlands; 4 Amsterdam Gastroenterology Endocrinology Metabolism, Amsterdam, The Netherlands; 5 Amsterdam Reproduction and Development research institute, Amsterdam, The Netherlands; 6 National Police of the Netherlands, Landelijke Eenheid, Centrum voor Biometrie, Zoetermeer, The Netherlands; 7 Dept. of Medical Biology, Amsterdam UMC location University of Amsterdam, Amsterdam, The Netherlands; 8 Dept. of Pediatric Surgery, Erasmus MC – Sophia Children’s Hospital, University Medical Center Rotterdam, Rotterdam, The Netherlands

**Keywords:** Postmortem fingerprinting, Micro-CT, Friction ridges, Poroscopy, Forensic identification, Decomposition

## Abstract

**Background:**

Traditional postmortem fingerprinting is often unreliable in cases of decomposition or thermal trauma. Although friction ridge patterns are typically recorded from the epidermis, their anatomical origin in the dermis may preserve identifiable structures, such as friction ridges and sweat pores, even when the skin’s outer layer is compromised. Minimally destructive postmortem imaging techniques capable of visualizing these subsurface structures could therefore greatly enhance forensic identification.

**Objective:**

This pilot study explores the feasibility of micro-computed tomography (micro-CT) as a novel, minimally destructive technique for visualizing subsurface fingerprint structures in challenging postmortem forensic contexts.

**Methods:**

Thirteen human finger samples in various postmortem conditions (fresh, decomposed and burned) were included. Four were scanned using micro-CT. Contrast staining was selectively applied. Micro-CT findings were descriptively contextualized using conventional postmortem fingerprinting techniques.

**Results:**

Micro-CT imaging successfully visualized friction ridges and sweat gland structures in fresh samples, including at lower scanning resolutions. In decomposed samples, micro-CT imaging revealed little anatomical detail, whereas warm water rehydration restored usable ridge patterns. In burned samples, micro-CT imaging failed to recover ridge detail but visualized sweat gland structures.

**Conclusion:**

Micro-CT imaging shows potential as a complementary tool for postmortem identification in burned or fresh remains but provides limited additional value in cases of advanced decomposition. As a pilot study, these findings primarily provide anatomical and technical insights. Future studies should explore larger sample sizes, sweat gland tracing and integration with 3D matching software.

**Supplementary Information:**

The online version contains supplementary material available at 10.1007/s00414-026-03760-x.

## Introduction

Fingerprint identification remains one of the most widely used and cost-effective biometric methods in forensic practice. However, in postmortem cases where decomposition or thermal trauma has compromised the epidermis, traditional fingerprint acquisition techniques, such as ink or powder methods, often prove ineffective [1]. This challenge raises a critical issue for forensic investigators: can fingerprint-based identification still be achieved when the skin’s outer layer is degraded or absent?

To answer this question, it is essential to understand the anatomical origins and resilience of fingerprint structures. Friction ridge patterns, which serve as the biometric foundation of fingerprint identification, are typically recorded from the epidermal surface [1]. However, the anatomical basis of these patterns originates deeper, within the dermal layer. The dermis contains both primary and secondary ridges; the latter reflect the fingerprint ridges visible on the skin surface, while the former house the terminal ducts of sweat glands [1]. Friction ridge patterns are not only anatomically layered but also biologically resilient and uniquely individual. Formed in utero beginning at the 10th week of gestation, these patterns are not only genetically determined but also anatomically resilient [1]. After superficial abrasion or removal of the stratum corneum, ridges tend to reappear in their original form, maintained by a structural renewal mechanism within the epidermis [2]. Even deliberate attempts at obliteration using dermabrasion, chemicals or burning have repeatedly failed to fully erase identifying features, underscoring the robustness of friction ridge anatomy. Only when damage extends deeper, beyond approximately 1 mm into the dermis, are these patterns permanently disrupted, typically resulting in scaring [3, 4]. Despite this resilience, forensic fingerprint preservation is time- and condition-dependent. Standard postmortem fingerprinting techniques are generally effective in the early postmortem period, but their reliability diminishes rapidly as decomposition advances. Environmental factors play a key role: in cold conditions, usable ridge detail may persist for several weeks, whereas in warm climates, autolysis, insect activity and skin slippage can render fingerprints unreadable within 2 to 4 days [5, 6].

Building on this anatomical foundation, attention has turned to imaging technologies that could capture subsurface structures. Among the candidate modalities, micro-computed tomography (micro-CT) has emerged as a promising minimally destructive technique. Micro-CT imaging is a high-resolution, non-destructive imaging modality capable of visualizing internal structures at micrometer scale [7]. Initially used in anatomical and material sciences, micro-CT imaging has gained traction in forensic contexts, including trauma evaluation and child abuse investigations [8–10]. Its ability to differentiate soft tissue contrasts and resolve minute anatomical detail suggests potential for visualizing subsurface ridge structures. Katsamenis et al. [11] demonstrated that micro-CT imaging can successfully reconstruct external ridge patterns from a fresh, intact cadaveric hand.

Micro-CT could provide crucial insights into burned samples where the epidermis is compromised and deeper dermal structures such as sweat glands should remain visible. This suggests a potential niche for micro-CT imaging in burned cases where traditional fingerprinting fails, as beyond ridge patterns, sweat pore morphology adds another layer of biometric individuality. First introduced by Locard in 1912, poroscopy focuses on the location, size and shape of sweat pores, which have been found to be relatively stable over time [12–16]. The potential to visualize these structures with micro-CT imaging has not been formally investigated, but if successful, it could greatly enhance identification in cases with epidermal loss. Given that sweat pores and sweat gland ducts reside in the dermis, we hypothesize that these features remain detectable via micro-CT imaging, even when the ridge patterns are lost due to decomposition or trauma.

Recovering such subsurface features is not only of technical interest, but also of operational importance. INTERPOL endorses three primary methods for human identification: DNA, dental records and dactyloscopy [17]. Dactyloscopy remains the most cost-effective and rapid biometric technique, provided viable ridge patterns can be recovered [18]. As international fingerprint databases continue to expand worldwide, improving postmortem fingerprint acquisition in difficult cases is of critical importance [18].

In this study, we evaluate the feasibility of micro-CT imaging as a novel, minimally destructive method for postmortem fingerprint identification in complex forensic scenarios to generate anatomical and technical insights and to inform future validation studies. We designed three experiments simulating different postmortem conditions to assess the diagnostic value of micro-CT imaging in capturing fingerprint structures. We aim to determine whether this technique can visualize dermal ridge structures and sweat gland remnants with sufficient resolution to support biometric identification where conventional methods are inadequate.

## Methods

This study comprised three experiments utilizing a total of thirteen isolated human fingers in varying postmortem conditions: fresh, decomposed, and laser-induced removal of superficial skin layers (hereafter referred to as ‘burned’ for readability). The specimens were obtained from three bodies donated to science through the Amsterdam UMC body donation program. The research protocol was reviewed and approved by the Biobank and Tissue Committee (BTC; approval number: 2024.0966). In addition, the Medical Ethics Review Committee (METC) of Amsterdam UMC issued a non-WMO declaration (notification number: 198130). An overview of sample characteristics is presented in Table 1. As this study was designed as a pilot feasibility study, one representative finger per experimental condition was selected for micro-CT imaging to explore anatomical visibility rather than diagnostic performance. A total of four fingers were scanned with micro-CT.

**Table 1:** Overview of sample characteristics. Information regarding the donor: gender, age and presence of decomposition: none, over 2 years or 5 days. Sample number: donor number-sample number), finger indicates which finger was used; varying from dig 1–5 of the left (L) or right (R) hand, applied additional techniques besides digital live scanning; ink, water, tissue builder or laser, staining duration in days (d) if applicable and whether the sample was scanned with micro-CT. - indicates no additional technique, staining or scanning was performed

Donor	Gender	Age (yr)	Decomposition	Sample	Finger	Additional Techniques	Staining duration	Micro-CT
1	Female	78	none	1.1	L3	-	10 d	Yes
				1.2	L4	ink	-	Yes
2	Male	87	> 2 yr	2.1	R3	-	6 d	Yes
				2.2	R4	warm water	-	-
				2.3	R5	tissue builder	-	-
				2.4	L2	warm water	-	-
				2.5	L3	warm water	-	-
				2.6	L5	warm water	-	-
3	Male	92	5 d	3.1	R2	laser	-	-
				3.2	R3	laser	-	-
				3.3	R5	laser	-	-
				3.4	L2	laser	-	-
				3.5	L3	laser	6 d	Yes

### Experiment 1: Fresh

Two fresh-frozen fingers were obtained from a fresh-frozen body and thawed. Traditional fingerprint acquisition methods were applied before fixation. Both samples were scanned using the digital live scanner: DERMALOG Fingerprint Scanner ZF 1 3rd generation (DERMALOG Identification Systems GmbH, Hamburg, Germany). One was additionally processed with ink and rolled onto paper to obtain a traditional fingerprint impression. The samples were fixed in 4% paraformaldehyde (PFA) for 48 h. One sample was stained with B-Lugol iodine solution for 10 days to enhance soft tissue contrast, while the other remained unstained and was stored in 2% PFA [19]. Prior to scanning, both samples were thoroughly rinsed with phosphate-buffered saline (PBS) and positioned in padded plastic containers to minimize motion artifacts during imaging. Micro-CT imaging was performed using the TESCAN UniTOM XL system (TESCAN, Brno, Czech Republic) by an experienced technician. The inked, unstained sample was scanned with micro-CT to assess baseline tissue contrast without enhancement. Multiple voxel resolutions were assessed to determine the optimal trade-off between image quality and scanning duration, as longer scans may delay forensic identification. Detailed scan parameters are listed in Table 2.

**Table 2:** Scan parameters. Resolution in voxelsize (micrometer), scantime in hours: minutes (h: m), used voltage in kilovolt (kV), power in Watt (W), exposure time in milliseconds (ms), number of projections and used Aluminium (Al) filter in millimeter (mm)

Sample	Voxelsize (µm)	Scantime (h: m)	Voltage (kV)	Power (W)	Exposure (ms)	Number of projections	Filter (mm)
1.1	7	2:58	160	15	3000	4283	1.0 Al
	12	4:09	160	15	3000	5000	1.0 Al
	20	0:15	160	20	400	2253	1.0 Al
	40	0:02	160	40	100	1127	1.0 Al
1.2	12	2:02	160	15	1500	5000	1.0 Al
	20	0:17	160	20	500	2064	1.0 Al
	40	0:02	160	40	140	1127	1.0 Al
2.1	10	1:30	160	15	1500	2700	1.0 Al
3.5	8	1:55	160	15	1500	2400	1.0 Al

### Experiment 2: Decomposed

Six samples were obtained from a human body which had been buried for over two years at the ARISTA human taphonomic research facility, Amsterdam UMC [20]. Initial fingerprint acquisition was attempted on all samples using the standard digital live scanner as the reference method. Additional traditional techniques were tested by two forensic police investigators. Four samples were used to explore and practice the rehydration process using the warm water immersion technique. The samples were exposed to a stream of warm tap water for a duration of 10 s. One sample was treated with tissue builder, a traditional embalming-derived technique intended to restore volume and ridge definition. This sample did not undergo further rehydration and was not selected for micro-CT imaging. One sample was fixed in 4% PFA, stained with B-Lugol iodine solution for six days (based on prior optimization) and subsequently imaged with micro-CT as described above.

### Experiment 3: Burned

Five fingers were obtained from the body and stored under refrigeration for five days. Thermal trauma was simulated using the Speedy 100 system (Trotec Laser GmbH, Marchtrenk, Austria), a laser engraving device provided by law enforcement. The aim was to expose the underlying dermal ridges. To optimize this process, various laser settings, specifically different depths and engraving speeds, were tested across the samples. Initial superficial removal of the epidermis using low-power laser settings did not adequately expose the ridge structures. Therefore, engraving mode was applied at 100% power (60 W) and 15% speed, repeated twice, to achieve deeper tissue removal. The effectiveness of epidermal removal was assessed visually and with the aid of a magnifying glass to determine whether sufficient dermal ridge detail had been exposed for further analysis. Although we refer to this condition as “burned,” the applied laser settings effectively vaporized superficial skin layers. This does not fully replicate thermal trauma but provides a controlled model for studying dermal ridge loss. All samples were scanned using the digital live scanner. One sample was selected for micro-CT imaging. This sample was fixed in 4% PFA, stained with B-Lugol iodine solution for six days and subsequently scanned using micro-CT as described in Experiment 1.

### Data analysis

All micro-CT data were processed using Amira software, version 3D 2021.2 (Thermo Fisher Scientific, Waltham, MA, USA), for image visualization and 3D volume rendering. Scans were visually assessed by a trained analyst, focusing on anatomical integrity and visibility of the epidermis, clarity and continuity of friction ridge patterns and detectability of sweat gland morphology.

## Results

### Experiment 1: Fresh

**Fig. 1:**
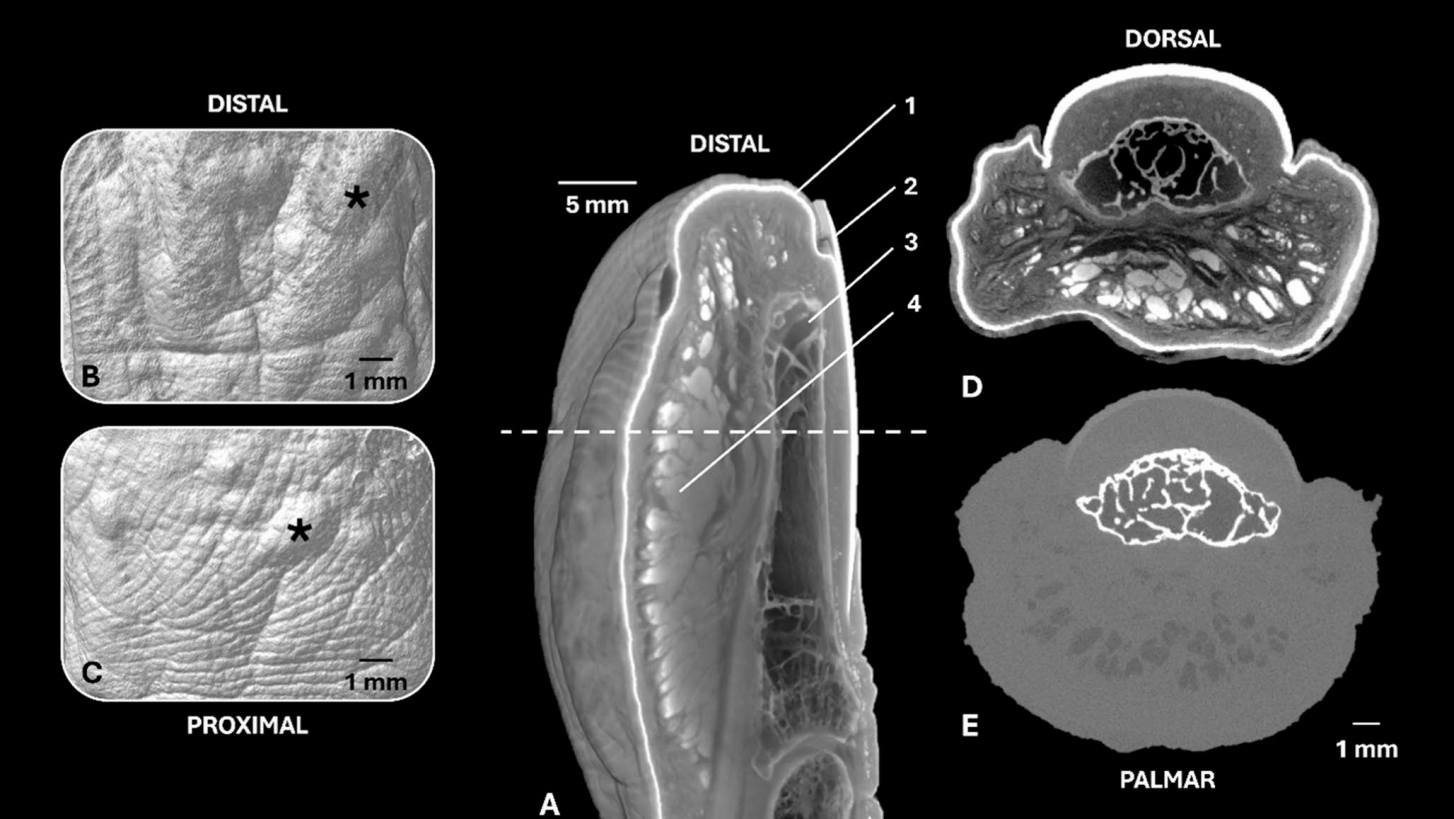
Comparison of the unstained and stained fresh samples micro-CT images (voxelsize 40 μm). **A** Ventrolateral view on a sagittal sliced 3D volume rendering model of sample 1.1 (stained) showing the external fingerprint in the front, hyperdense skin, nail, bone and soft tissues in the slice and an indication of the level of the transverse sections in D-E *(dotted line)*. **B** External fingerprint of 3D volume rendering model of sample 1.1 (stained) showing ridges and pores *(asterisk)*. **C** External fingerprint of 3D volume rendering model of sample 1.2 (unstained) showing ridges and bumps on the skin *(asterisk)*. **D** Transverse section of sample 1.1 (stained) showing the hyperdense nail, skin and soft tissues. **E** Transverse section of sample 1.2 (unstained) showing hyperdense bone tissue. (1) Epidermis. (2) Nail. (3) Distal phalanx bone. (4) Subcutaneous tissue

**Fig. 2:**
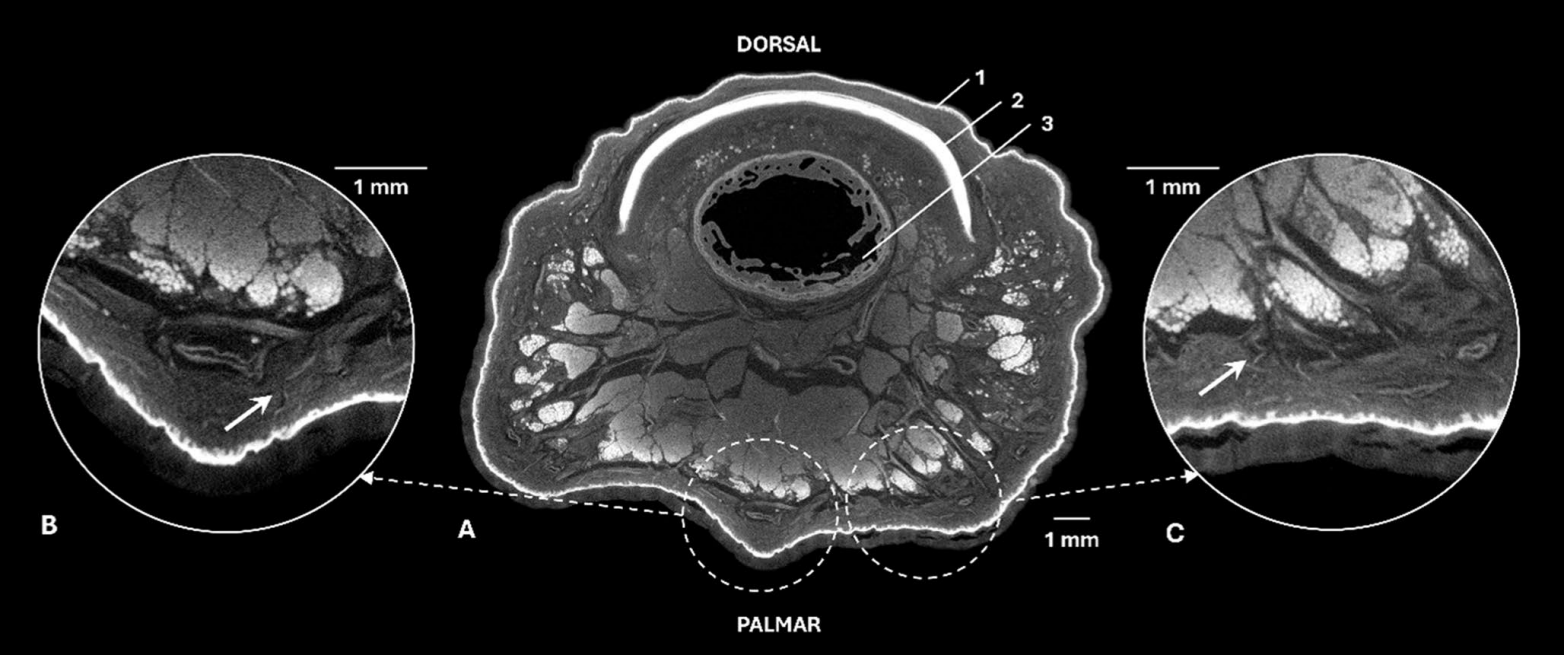
Detailed transverse section of the stained fresh sample micro-CT images (voxelsize 7 μm). **A** Transverse section of sample 1.1 showing hyperdense skin, nail and soft tissues. **B** Zoomed-in region of image A showing ridges and sweat glands *(arrow)*. **C** Another zoomed-in region of image A showing ridges and sweat glands *(arrow)*. (1) Epidermis. (2) Nail. (3) Distal phalanx bone

Conventional fingerprint acquisition using both ink and a digital live scanner yielded identifiable ridge patterns. Based on sample size, staining of sample 1.1 with B-Lugol was completed within ten days. Micro-CT imaging was successfully performed on both samples, testing a range of voxel sizes (7–40 μm) and scanning durations (2 min to 4 h). A rapid 2-minute scan at 40 μm resolution of the unstained sample (1.2) was sufficient to visualize identifiable fingerprint structures (Fig. 1). High-resolution micro-CT imaging at 7 μm of the stained sample (1.1) allowed clear visualization of both primary and secondary ridges, as well as internal anatomical features such as vascular structures and sweat glands (Fig. 2). In addition, several bumps on the surface of the skin were visually detected throughout the fingertip of the samples.

### Experiment 2: Decomposed

**Fig. 3:**
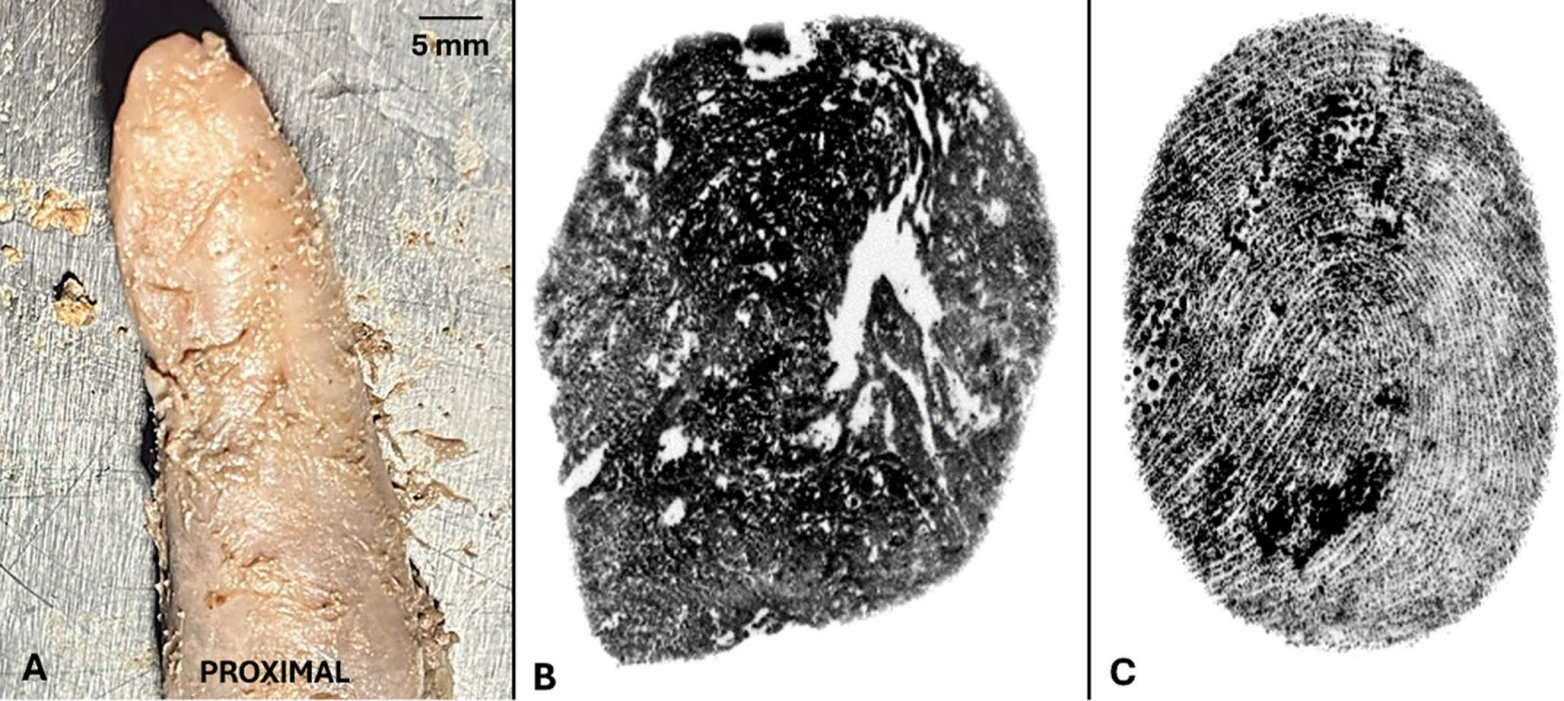
Digital live scan images of one decomposed sample, before and after the warm water method. **A** Photo of the palmar side of the distal and middle phalanx of sample 2.5, showing no visible external fingerprint. **B** Digital live scan image of sample 2.5 before applying any additional techniques, showing no usable print. **C** Digital live scan image of sample 2.5 after applying the warm water method, showing a usable print

**Fig. 4:**
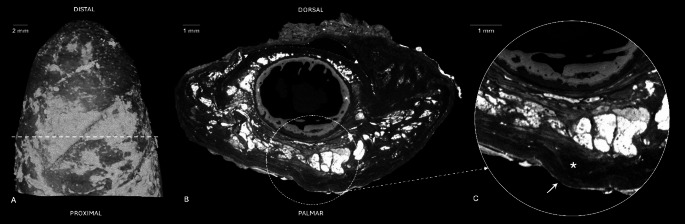
Micro-CT images of the decomposed sample (voxelsize 10 μm). **A** 3D volume rendering model of sample 2.1 showing no external fingerprint and indicating the level of the transverse section in B-C (dotted line). **B** Transverse section of sample 2.1 showing hyperdense soft tissues. **C** Zoomed-in region of image **B** showing loss of the (epi)dermis *(arrow)*, no discernible ridges and degraded vascular and glandular structures *(asterisk)*

The decomposed fingers showed epidermal slippage, tissue fragility and visual absence of intact skin layers (Fig. 3). Standard digital live scanning prior to any intervention yielded no usable fingerprints. After the injection of tissue builder, ridge detail remained insufficient for identification. However, after rehydration using a warm water immersion method, usable fingerprints could be acquired with the digital live scanner (Fig. 3). Sample 2.1 was completely stained in six days, which was followed by micro-CT imaging at a voxel size of 8 μm. This resolution was selected as the best achievable resolution based on the sample’s size. Scan images revealed complete loss of the epidermis. No ridge patterns were discernible and vascular and glandular structures were severely degraded, likely due to advanced tissue decomposition (Fig. 4).

### Experiment 3: Burned

**Fig. 5:**
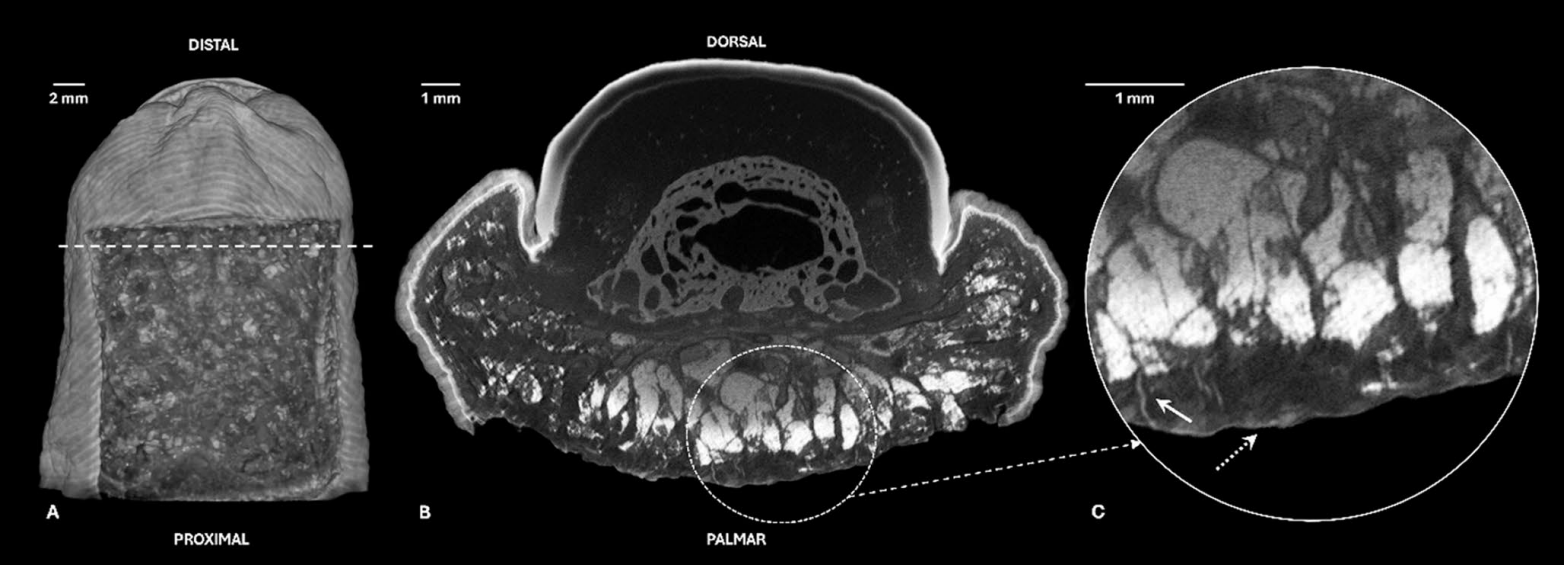
Micro-CT scan images of the burned sample (voxelsize 8 μm). **A** 3D volume rendering model of sample 3.5 showing no external fingerprint at the lasered surface and indicating the level of the transverse section in B-C (dotted line). **B** Transverse section of sample 3.5 showing hyperdense skin, nail and soft tissues. **C** Zoomed-in region of image B showing loss of the (epi)dermis *(dotted arrow)* and preserved vascular and glandular structures *(arrow)*

Digital live scanning failed to detect identifiable fingerprint patterns. Sample 3.5 was subsequently completely stained in six days and scanned using micro-CT at 10 μm resolution, the highest achievable resolution for the sample size. Scan images revealed that the laser had penetrated deeper than anticipated, extending into the dermal layer. Consequently, ridge structures were no longer present. Nonetheless, vascular and sweat gland structures remained partially intact and traceable, offering promising avenues for structural reconstruction (Fig. 5).

## Discussion

This pilot study explores the feasibility and potential of micro-CT imaging as a method for postmortem fingerprint visualization under varying forensic conditions. Each experimental condition yielded unique insights, which are discussed below.

### Experiment 1: Fresh

Based on our experience, the application of B-lugol staining combined with micro-CT scanning proved to be practically feasible and required no complex adjustments. The micro-CT scans provided high-resolution visualizations of both friction ridge patterns and internal anatomical structures, including sweat glands (Fig. 2). These results confirm previous findings on the anatomical value of micro-CT imaging and demonstrate its capacity for detailed, internal fingerprint imaging. Such high-quality visualizations are not only promising for anatomical research and forensic education but may also have direct forensic relevance. In addition to ridge and gland morphology, micro-CT enables verification of the normal continuity of dermal and epidermal ridge structures. This ability could be particularly valuable in rare cases of attempted fingerprint manipulation, such as surgical transplantation of friction ridge skin, where scans may reveal unnatural discontinuities or irregular layering, features that are difficult to detect with conventional 2D fingerprinting techniques.

In addition, micro-CT imaging revealed localized detachment of the epidermis and the presence of discrete, dome-shaped surface elevations, primarily on the volar aspect of the distal phalanges (Fig. 1). These alterations were exclusively observed in fresh-frozen samples, suggesting cryo-induced disruption of skin integrity due to ice crystal formation and subsequent osmotic stress during freezing and thawing [21]. These processes are consistent with the observed partial lifting of the epidermis in affected specimens. The small, rounded elevations observed on the fingerprint ridges may correspond anatomically to the sweat pores, which are densely distributed on the volar surface of the fingertips. Each friction ridge contains sweat ducts that terminate at the primary ridge apex [22, 23]. Disruption of the intraepidermal duct segment, by ice crystal formation or localized pressure during thawing, could lead to focal swelling or fluid retention, manifesting as surface protrusions. The distribution pattern of these features supports this interpretation. While similar effects were not detected on other parts of the sample, the volar skin’s specialized structure, characterized by a thick stratum corneum, pronounced rete ridges and high sweat gland density, likely increases its vulnerability to morphological changes following cryopreservation [23, 24]. Although histological analysis was beyond the scope of this study, such an approach could further elucidate the underlying microstructural alterations revealed by imaging.

### Experiment 2: Decomposed

Based on visual assessment, forensic police investigators anticipated no viable outcomes from conventional forensic techniques. However, after rehydration with the warm water method, one finger was successfully scanned using a digital live scanner, resulting in a usable fingerprint (Fig. 3). This supports previous reports on the effectiveness of warm water immersion [25, 26], and our findings indicate that this technique may offer potential for further optimization. Fields and Molina [25] demonstrated that rehydration under warm running water for 10–15 min and massaging the finger pad, could restore sufficient ridge detail to obtain usable postmortem fingerprints. Similarly, Uhle and Leas [26] described the boiling technique, in which deteriorated ridge skin is immersed in near-boiling water for 5–10 s to achieve temporary rehydration and pliability. In our case, although the application of warm running water was brief (10 s) and did not include massaging the tissue, it yielded usable ridge detail. This suggests that even minimal exposure may be effective in certain conditions and highlights the potential for further refinement and case-specific adaptation of existing rehydration protocols. In standard practice, investigators would not have attempted this approach, but this outcome may encourage its consideration in future cases involving advanced decomposition. The tissue builder technique, described in The Fingerprint Sourcebook [1], was attempted on a single sample but did not produce a usable fingerprint using the digital live scanner. While it has been noted as potentially useful in certain cases of desiccation or mummification, its effectiveness in advanced decomposition is limited, particularly when epidermal separation or severe dermal deterioration is present [1].

Notably, micro-CT imaging did not provide additional forensic value in the context of advanced decomposition (Fig. 4). While histological changes associated with prolonged postmortem intervals, such as tissue disintegration, dermal–epidermal separation, and disruption of sweat gland morphology, are well documented in the literature [27, 28], our findings offer direct three-dimensional visualization of these processes in situ. The micro-CT images revealed extensive structural reorganization, emphasizing that once such alterations have occurred, even high-resolution imaging techniques like micro-CT are unlikely to yield usable forensic ridge information. Nevertheless, micro-CT may retain value for addressing other research questions, such as investigating decomposition dynamics or tracing internal features in less degraded specimens. Furthermore, this experiment demonstrated that a staining duration of approximately six days is sufficient for whole adult human fingers, providing a practical reference for future studies employing contrast-enhanced micro-CT.

### Experiment 3: Burned

Burn injury simulation via laser proved difficult to control and assess, as we lacked pre-scan depth measurements to determine the extent of tissue damage prior to imaging. The scan data revealed that the laser had penetrated deeper than anticipated, extending into the dermal layer and resulting in the loss of ridge patterns (Fig. 5). However, vascular and sweat gland structures remained partially preserved and traceable, suggesting that despite ridge loss, deeper dermal architecture may still offer potential for structural reconstruction (Fig. 5).

These findings may inspire further research into poroscopy [12–16]. While sweat pores are less stable than ridge patterns or minutiae, they can provide additional value in cases where primary ridge detail is compromised. Research shows that features like pore inter-distance and position may support individualization [12, 14, 15]. However, studies also highlight challenges such as variable pore visibility, sensitivity to distortion and lack of standardization in analysis methods. Notably, Monson et al. [12] concluded that pore characteristics are not strictly permanent or persistent, limiting their standalone forensic reliability. Nonetheless, as originally suggested by Locard and later supported by biometric developments, poroscopy may serve as a useful complementary tool, especially in degraded or partial prints. The visibility of sweat glands in micro-CT data also corresponds with results from our fetal imaging study, where successful segmentation of sweat glands has been achieved [unpublished data]. Although labor-intensive, such segmentation may offer a pathway for reconstructing partial fingerprints, even in cases where ridge detail is no longer externally visible. At present, visualization of sweat gland structures should be regarded as descriptive anatomical information rather than a validated means of identification.

In future research, sweat glands could be segmented and 3D reconstructed from micro-CT scans. If their trajectories can be traced back to the ridge surface, it may enable digital reconstruction of a usable fingerprint, potentially matching it to entries in forensic databases. Integrating 3D micro-CT imaging with dactyloscopic software would be a critical next step, as current fingerprint identification tools are limited to 2D inputs. This also opens the door for combining micro-CT with other modalities such as optical coherence tomography (OCT) and immunolabeling. OCT is a non-destructive imaging technique that generates high-resolution cross-sectional images of biological tissues based on light scattering properties [29]. It has shown particular promise in visualizing internal fingerprints located at the epidermal-dermal junction, which may remain preserved even when surface ridges are lost due to decomposition [29]. Immunolabeling, on the other hand, uses targeted antibodies to bind specific tissue structures, such as sweat glands or connective tissue and can enhance image contrast at the microanatomical level [30]. When combined with high-resolution imaging techniques like micro-CT, immunolabeling may help delineate fine glandular and ridge structures more clearly [30]. Such multimodal approaches could improve both the quality and interpretability of forensic fingerprint evidence in severely compromised cases.

### Limitations and implications

The primary limitations of this study include its small sample size and pilot nature. Only one sample per experimental condition was scanned, preventing comparisons or statistical analysis. Furthermore, no trained fingerprint analysts were involved, as no experts are currently trained to evaluate fingerprints obtained through micro-CT imaging. Quantitative performance metrics such as sensitivity, specificity or inter-observer agreement were beyond the scope of this pilot study, as these require larger sample sizes and trained evaluators. Additionally, no fingerprint reconstruction or matching procedures were included, as current official police software is limited to 2D fingerprint analysis. Nevertheless, dactyloscopic experts who evaluated our 3D micro-CT scans indicated that these images could already be used as forensic evidence. Their application is currently less efficient due to the absence of dedicated software for converting or analyzing 3D fingerprint data. Conceptually, future developments could include digital flattening of the 3D dataset or the production of a 3D-printed model, from which a conventional 2D fingerprint impression might be obtained, potentially allowing integration with existing 2D fingerprint databases.

In addition to these methodological constraints, our findings also highlight several practical considerations relevant for forensic application. Most notably, ridge detail could be visualized in fresh samples even without contrast enhancement (Fig. 1), suggesting that in time-critical scenarios, rapid scanning without staining may already yield valuable results when intact fingers are available. Contrast staining, on the other hand, clearly adds value when deeper dermal features such as sweat glands are of interest. From an operational perspective, shorter staining protocols would be preferable, as faster procedures directly translate into quicker identification. Full penetration of contrast into the tissue core may not be required when ridge recovery is the primary aim, which can be achieved with shorter staining duration. Rather than limitations, these insights represent practical lessons that can inform the optimization of micro-CT protocols for specific forensic scenarios.

Despite these challenges, this study represents the first instance of capturing a finger at this resolution using (contrast-enhanced) micro-CT. Our results support the further exploration of micro-CT imaging as a complementary forensic tool, particularly for sweat gland trajectories in burned remains. Although DNA analysis, dental comparison and dactyloscopy are all well-established methods for human identification, the continued global expansion of fingerprint databases [18] highlights the value of developing non-destructive techniques for fingerprint recovery, particularly in disaster victim identification and complex forensic cases.

## Conclusion

This pilot study demonstrates that micro-CT imaging can be a promising technique for visualizing subsurface fingerprint structures in postmortem forensic cases, particularly when traditional methods fail due to thermal trauma or superficial epidermal loss. However, its forensic value appears limited in severely decomposed remains, where conventional rehydration methods are more effective. Future validation studies should include larger sample sizes and involve trained fingerprint analysts to assess diagnostic performance. Future research should focus onexploring sweat gland segmentation and integrating micro-CT data into existing forensic fingerprint identification software.

## Supplementary Information

Below is the link to the electronic supplementary material.

Supplementary Material 1.

## Data Availability

The datasets generated and/or analyzed during the current study are available from the corresponding author on reasonable request.
