# Supplementary material for: Exploring micro-CT as a novel approach for postmortem fingerprint identification
Source: Int J Legal Med. 2026 Mar 9;140(4):2143–52. doi: 10.1007/s00414-026-03760-x (PMC13275565; doi:10.1007/s00414-026-03760-x)
Supplement: Supplementary file 1 — Supplementary Material 1. [file 414_2026_3760_MOESM1_ESM.docx]

Exploring Micro-CT as a Novel Approach for Postmortem Fingerprint Identification

*International Journal of Legal Medicine*

Greta M.M. Timmerman^1,2*^, Daniel Docter^1,3,4,5^, Bart L.M. Kraus^6^, Jermo Hanemaaijer-van der Veer^1^, Roelof-Jan Oostra^5,7^, Bernadette S. De Bakker^1,5,8^

1. Amsterdam UMC location University of Amsterdam, Dept. of Obstetrics and Gynecology, Amsterdam, The Netherlands.
2. Netherlands School of Public & Occupational Health (NSPOH), Forensic Medicine, Utrecht, The Netherlands.
3. Amsterdam UMC, location AMC, Dept. of Pediatric Surgery, Emma Children’s Hospital, Amsterdam, The Netherlands.
4. Amsterdam Gastroenterology Endocrinology Metabolism, Amsterdam, The Netherlands.
5. Amsterdam Reproduction and Development research institute, Amsterdam, The Netherlands.
6. National Police of the Netherlands, Landelijke Eenheid, Centrum voor Biometrie, Zoetermeer, The Netherlands.
7. Amsterdam UMC location University of Amsterdam, Dept. of Medical Biology, Amsterdam, The Netherlands.
8. Erasmus MC – Sophia Children’s Hospital, University Medical Center Rotterdam, Dept. of Pediatric Surgery, Rotterdam, The Netherlands.

#### *Corresponding author: [g.m.timmerman@amsterdamumc.nl](mailto:g.m.timmerman@amsterdamumc.nl)

ORCID ID
Timmerman GMM: 0009-0000-0633-4536
Docter D: 0009-0004-9650-877X
Hanemaaijer-van der Veer J: 0009-0006-1391-2668
Oostra RJ: 0000-0002-2452-8307

De Bakker BS: 0000-0003-0758-4064
